# Supplementary figures and images for: Pan-genome and resistome analysis of extended-spectrum ß-lactamase-producing Escherichia coli: A multi-setting epidemiological surveillance study from Malaysia
Source: PLoS One. 2022 Mar 10;17(3):e0265142. doi: 10.1371/journal.pone.0265142 (PMC8912130; doi:10.1371/journal.pone.0265142)

# BUSCO Assessment Results

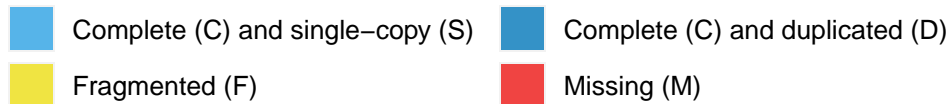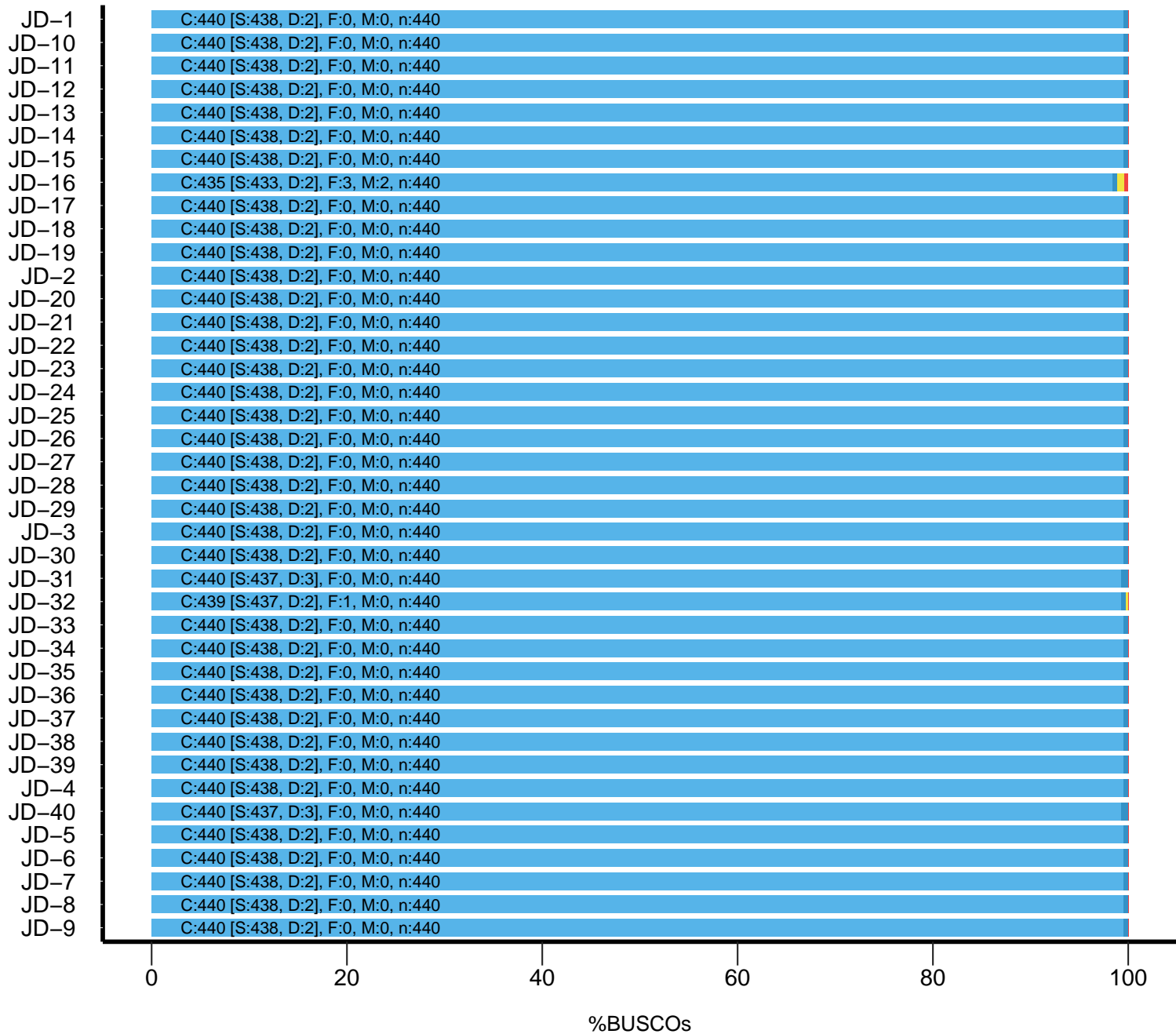

Supplement: S1 Fig — (PDF) [file pone.0265142.s001.pdf]

**a**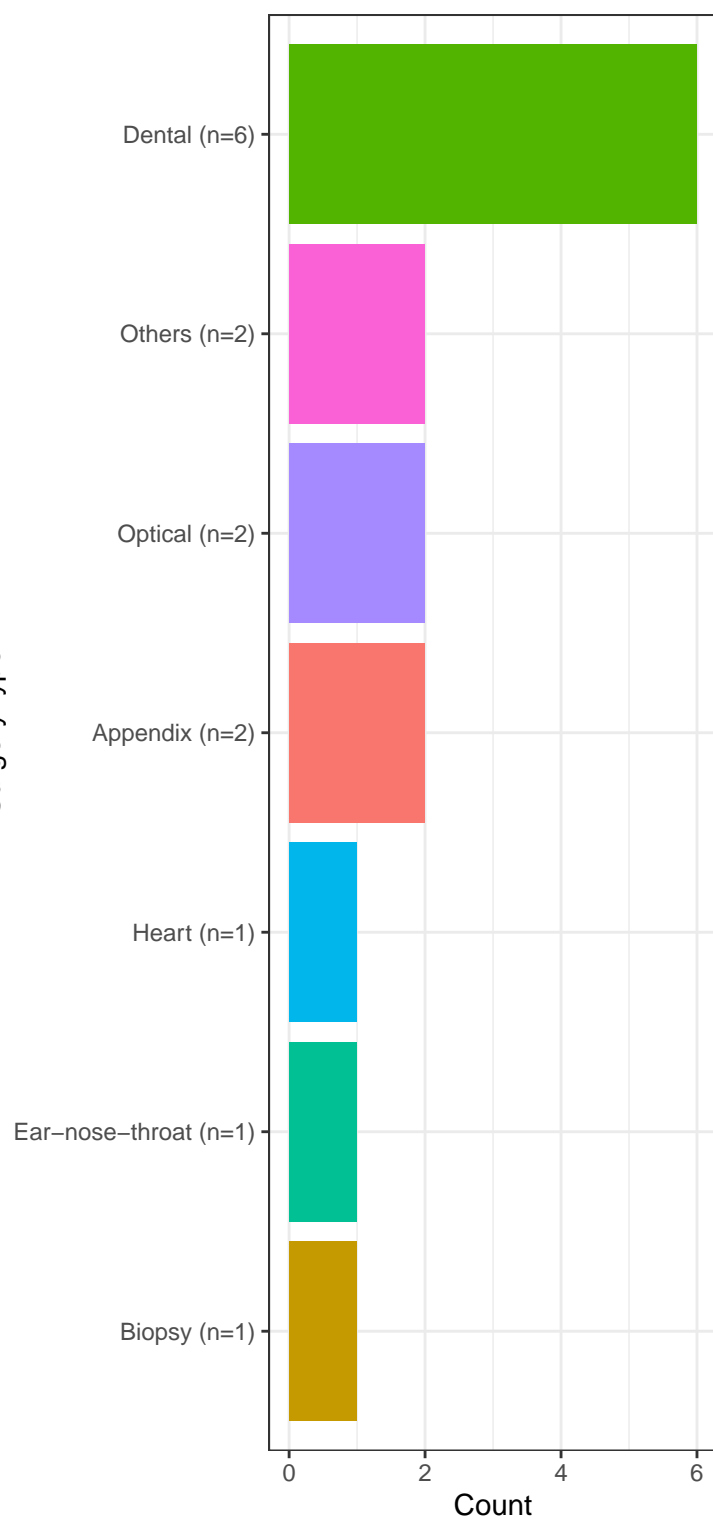**b**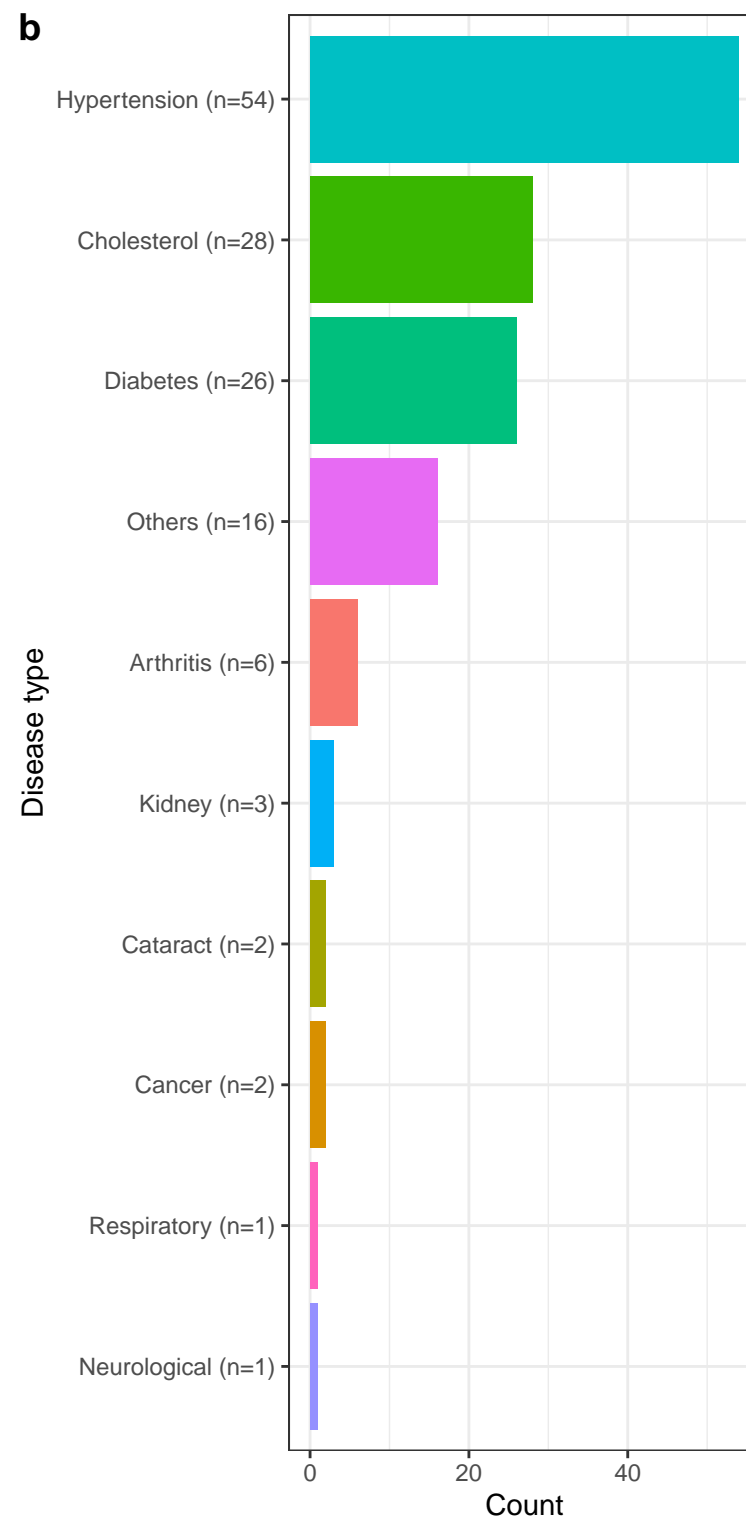**c**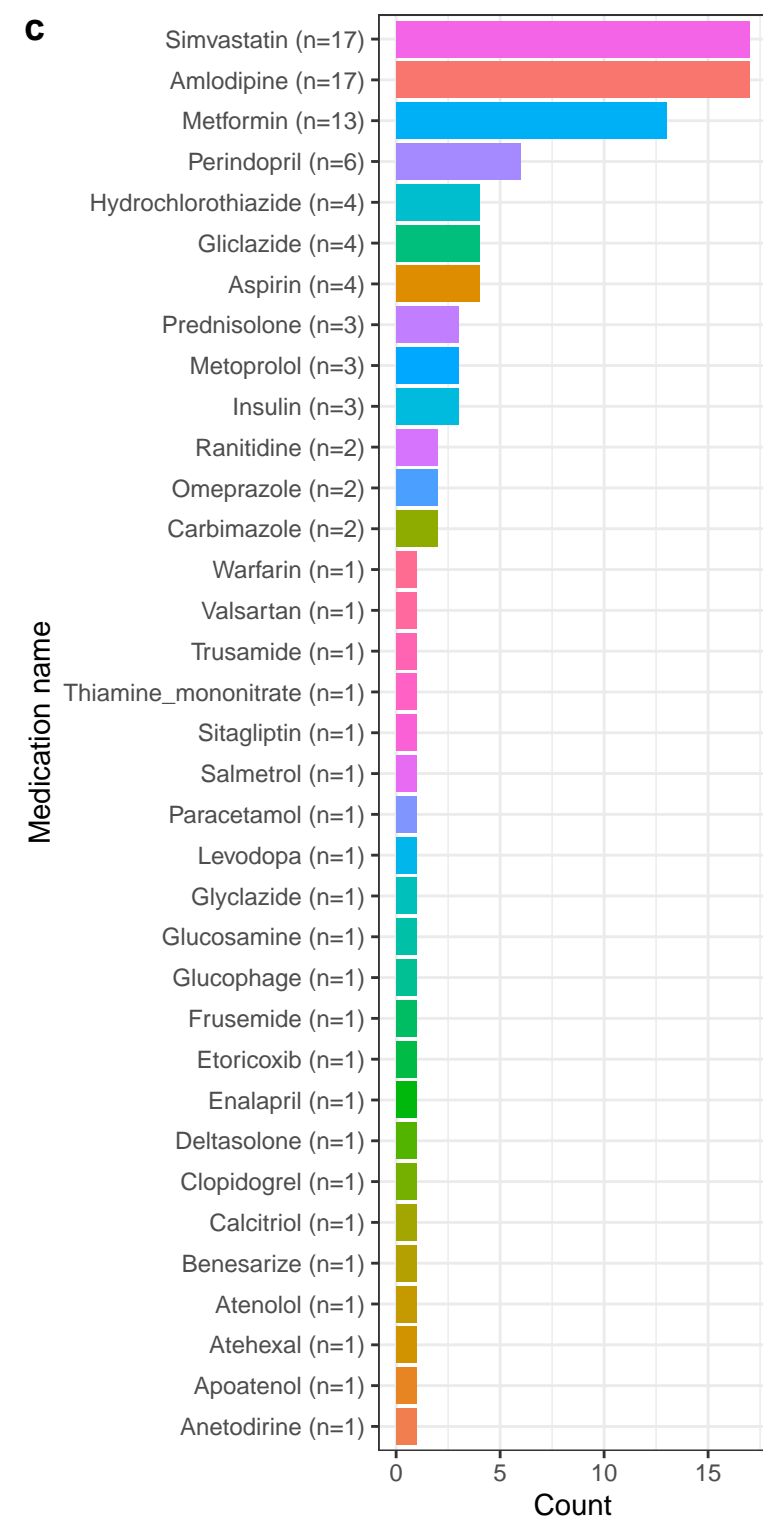

Supplement: S2 Fig — Health demographic data of the subjects included in the study, including (a) surgical history in the past year, (b) comorbidities, (c) and active medication. (PDF) [file pone.0265142.s002.pdf]

Clinical

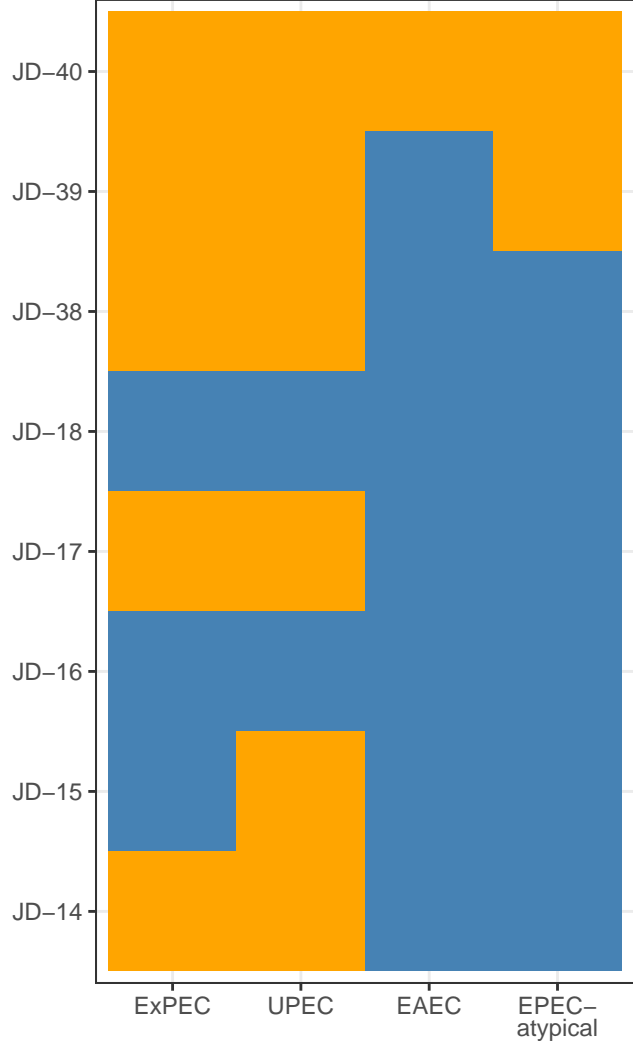

Community

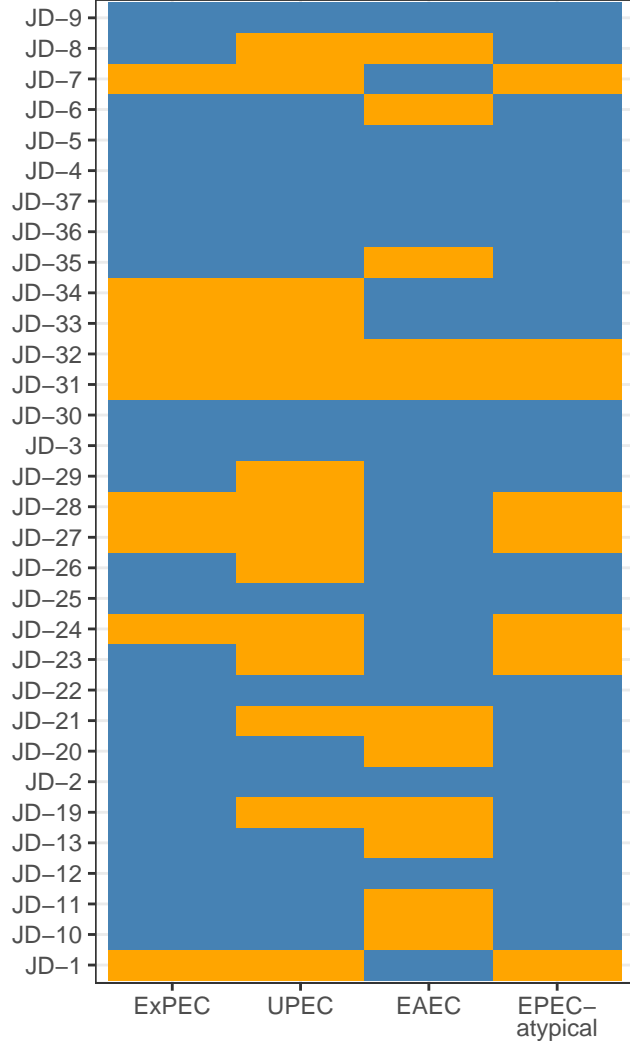

Negative  
Positive

Supplement: S3 Fig — (PDF) [file pone.0265142.s003.pdf]

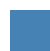

Higher in clinical isolates

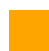

Higher in community isolates

Virulence Gene

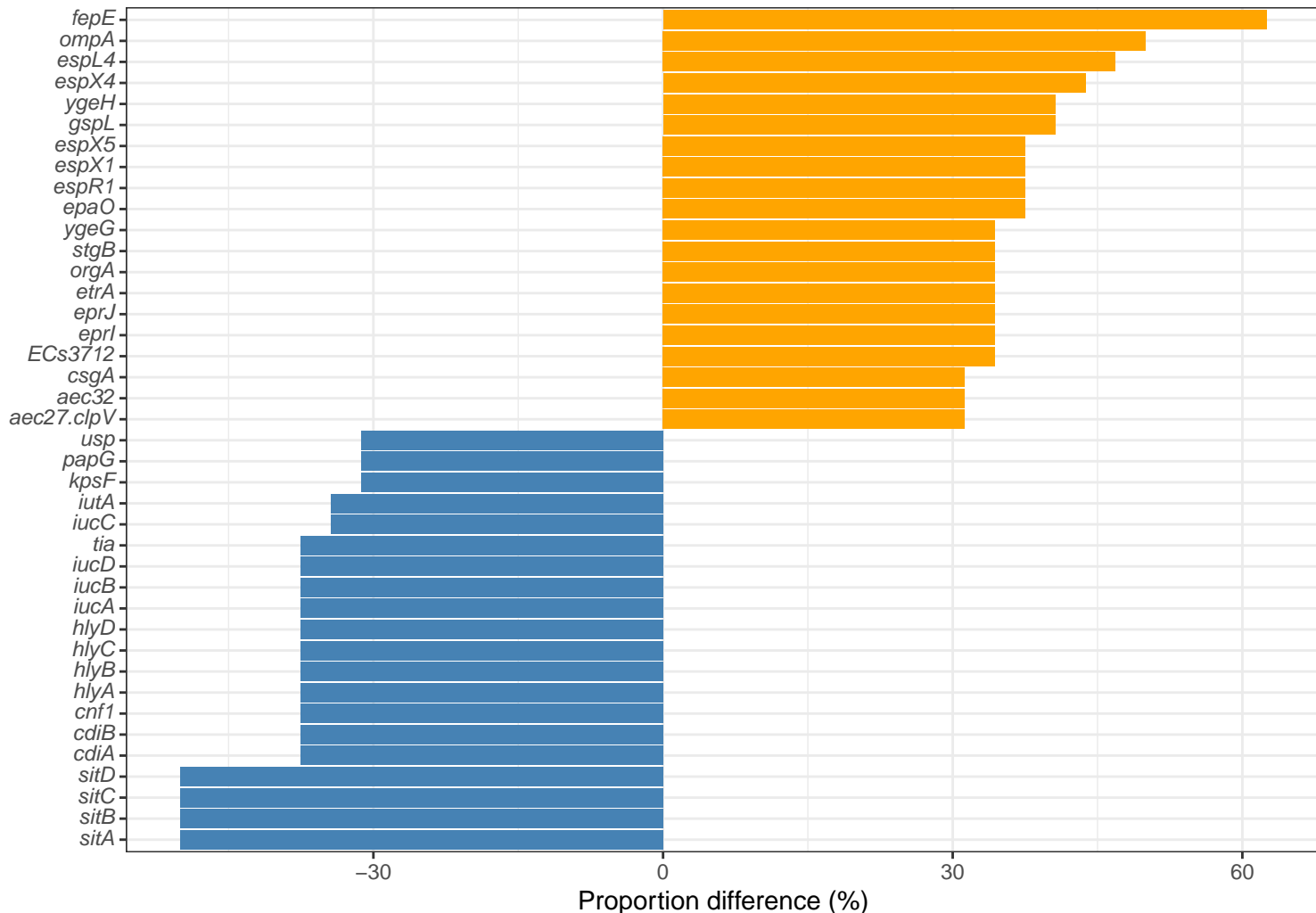

Supplement: S4 Fig — (PDF) [file pone.0265142.s004.pdf]

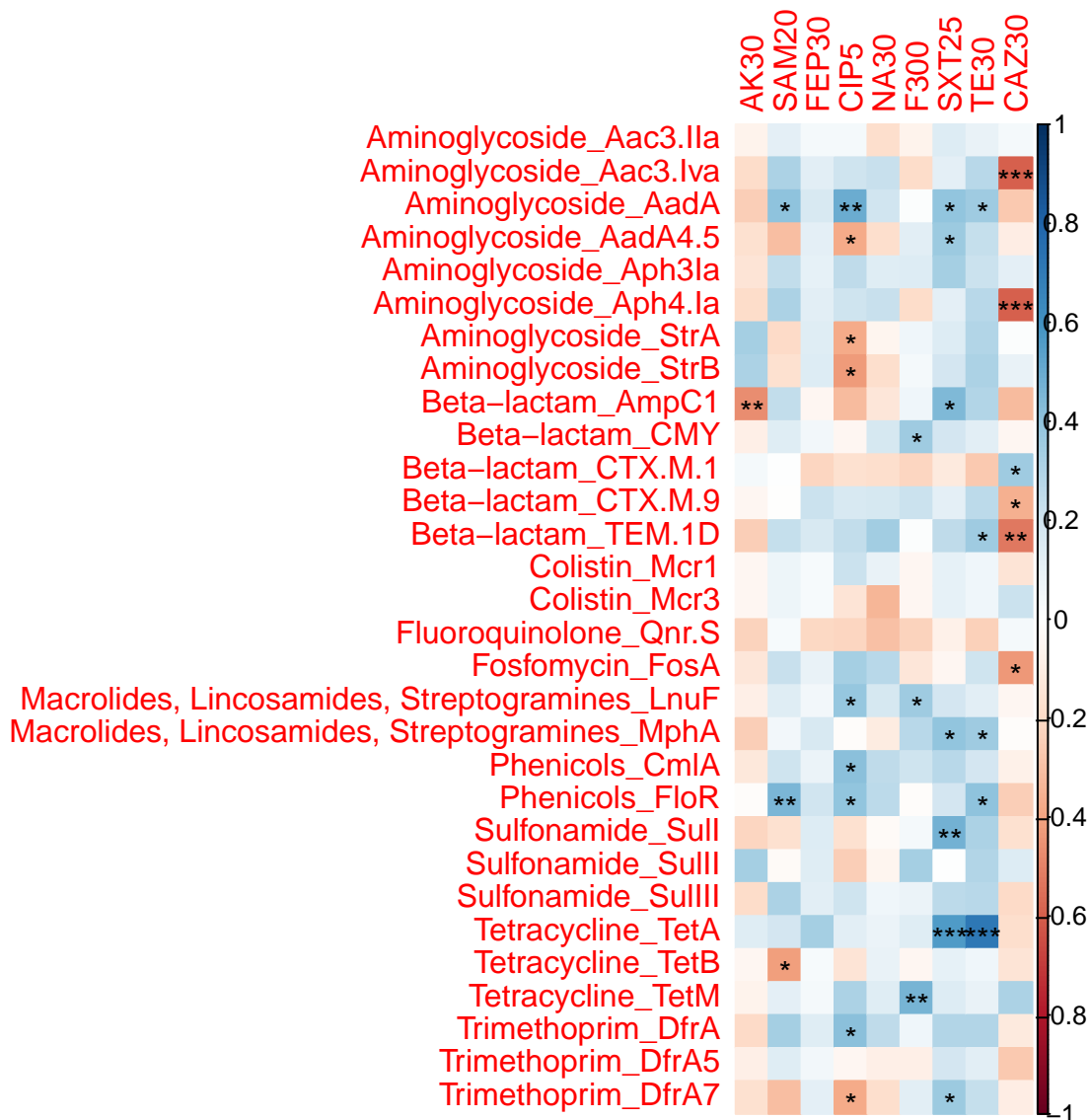

Supplement: S5 Fig — (PDF) [file pone.0265142.s005.pdf]

Samples

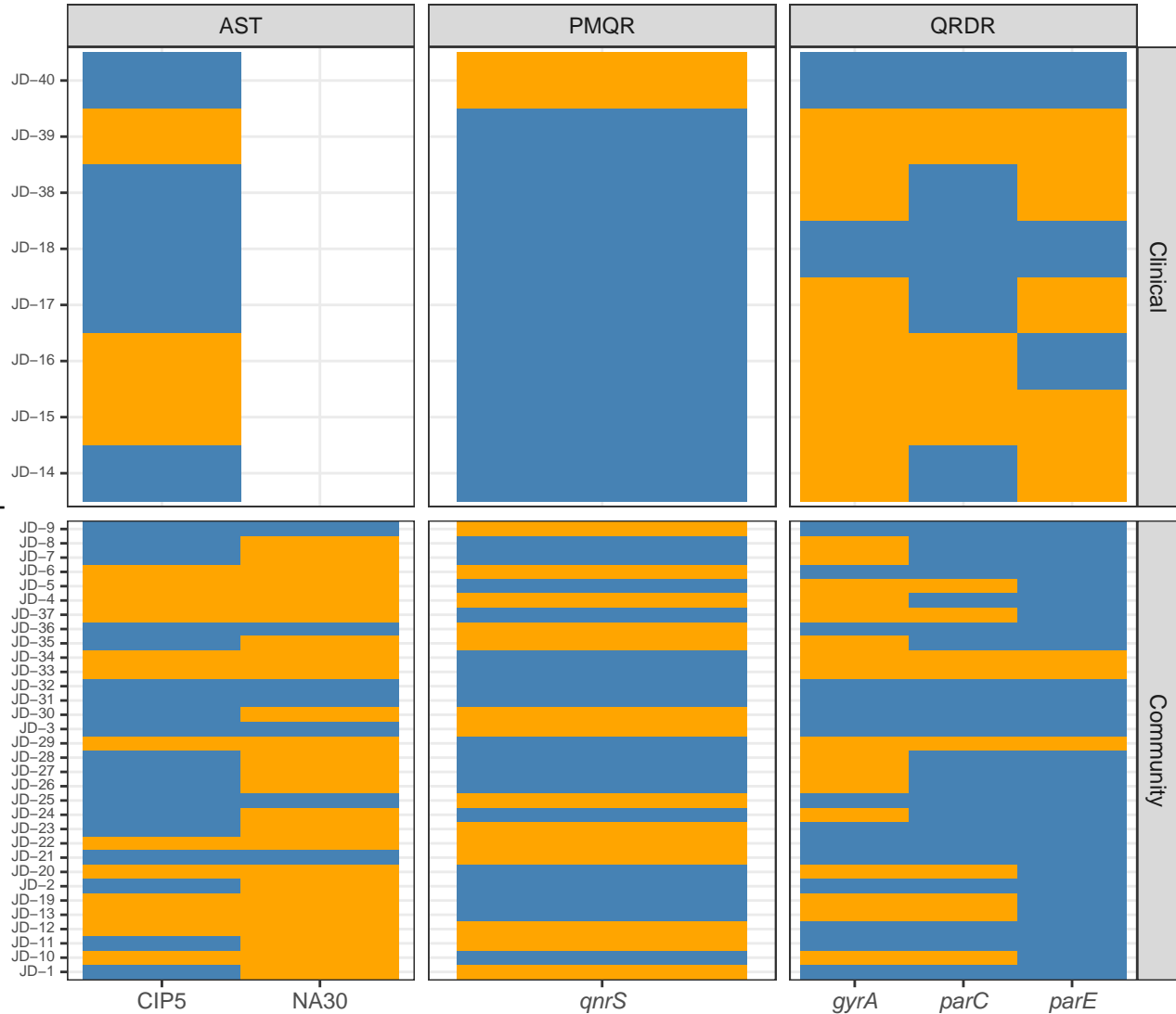

Supplement: S6 Fig — Abbreviations: CIP5 = Ciprofloxacin (5 μg); NA30 = Nalidixic Acid (30 μg). (PDF) [file pone.0265142.s006.pdf]

CTX-M variant

|            |            |            |
|------------|------------|------------|
| ● CTX-M-15 | ● CTX-M-3  | ● CTX-M-65 |
| ● CTX-M-27 | ● CTX-M-55 | ● CTX-M-69 |

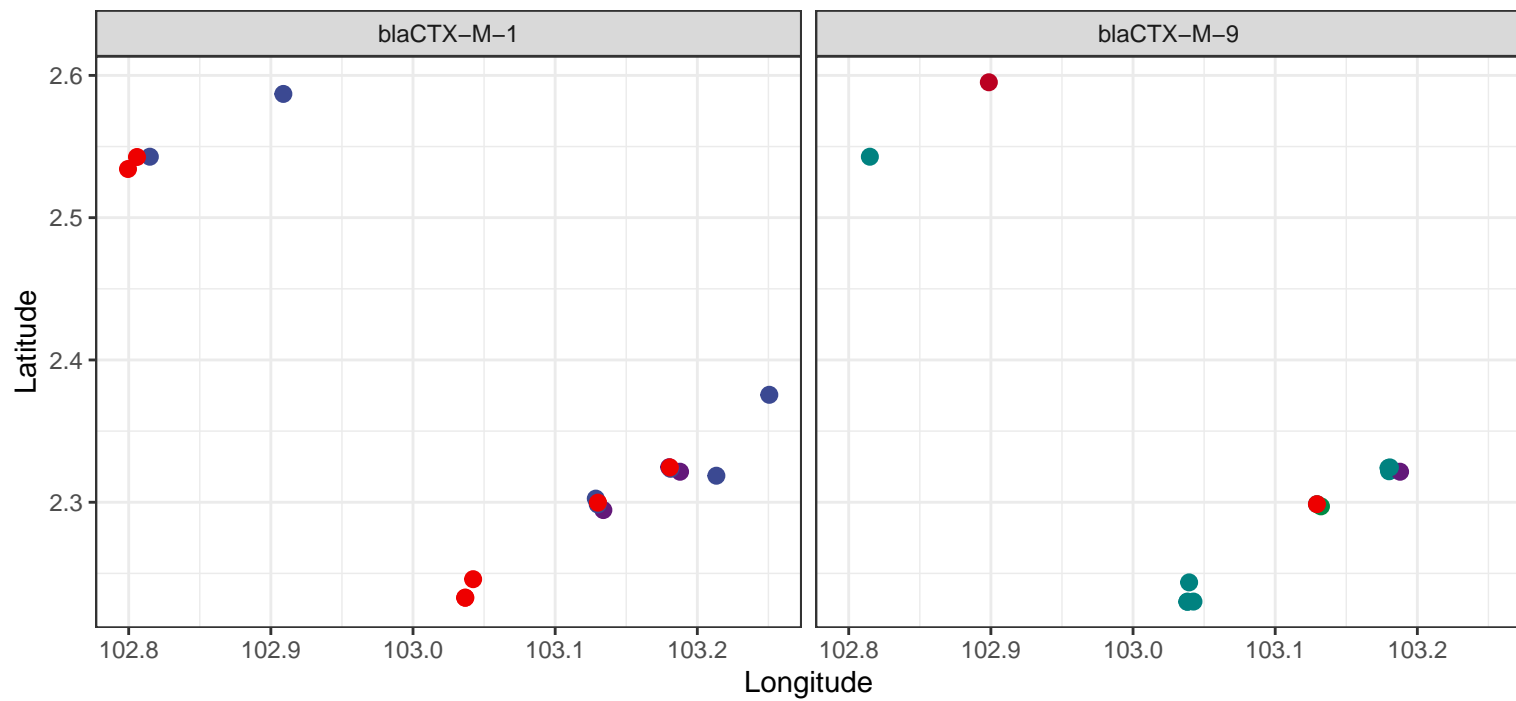

Supplement: S7 Fig — (PDF) [file pone.0265142.s007.pdf]

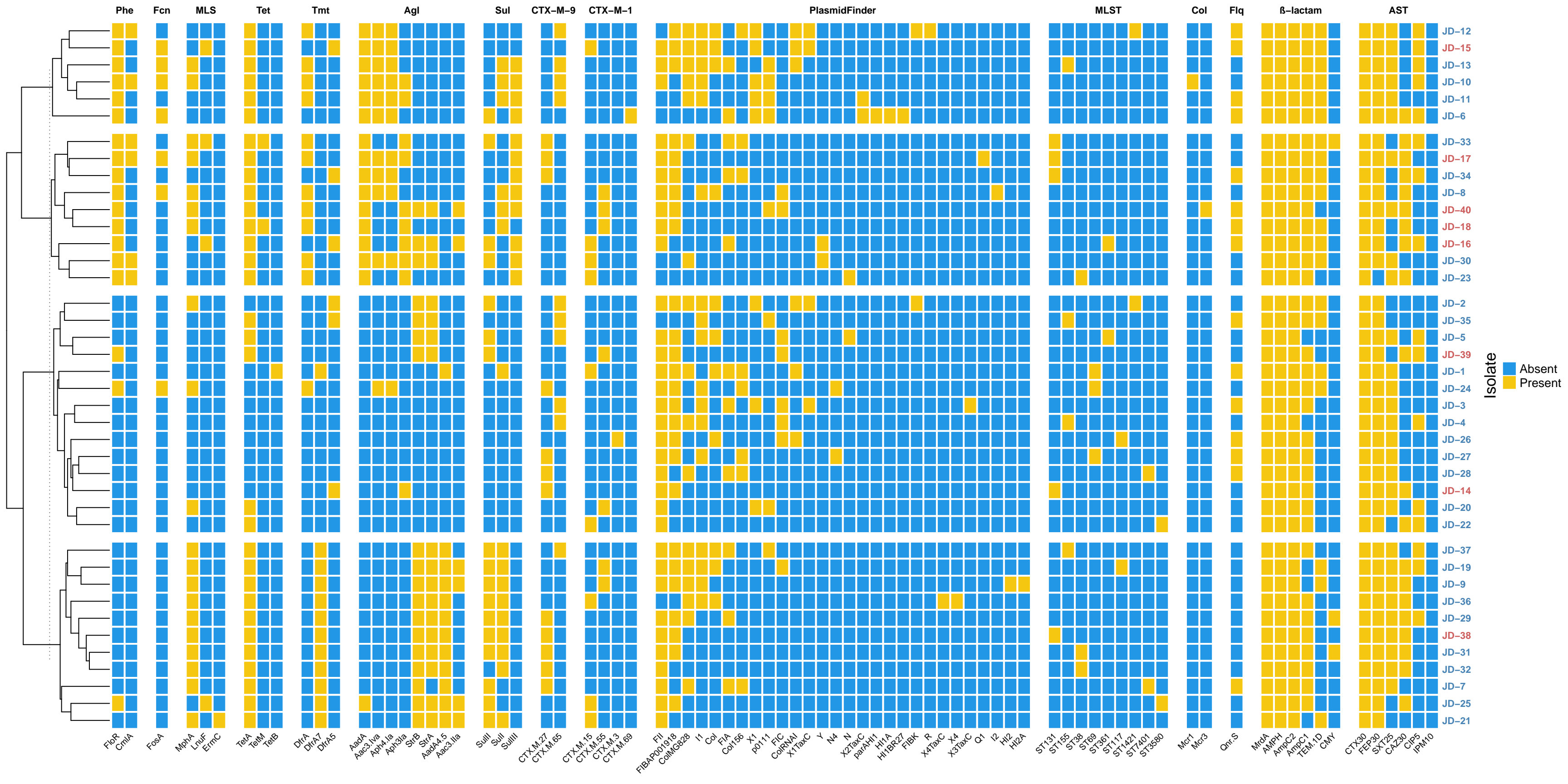

Supplement: S8 Fig — Community isolates are labelled in blue, while clinical isolates are labelled in red. Abbreviations: Fcn = fosfomycin; Phe = phenicols; MLS = macrolides and lincosamides; Tet = tetracycline; Agl = aminoglycoside; Tmt = trimethoprim; Col = colistin; Flq = fluoroquinolone; AST = Antibiotic susceptibility profile. (PDF) [file pone.0265142.s008.pdf]
